# The quality of German - language patient decision aids for oncological patients on the internet

**DOI:** 10.1186/s12911-023-02259-4

**Published:** 2023-08-18

**Authors:** Julia Möller, Lena Josfeld, Christian Keinki, Nathalie Zieglowski, Jens Büntzel, Jutta Hübner

**Affiliations:** 1 Klinik für Innere Medizin II Hämatologie und Internistische Onkologie, Universitätsklinikum Jena, Am Klinikum 1, 07747 Jena, Germany; 2 Klinik für HNO-Erkrankungen Kopf-Hals-Chirurgie, Interdisziplinäre Palliativstation Südharz Klinikum Nordhausen, Dr.-Robert-Koch-Straße 39, 99734 Nordhausen, Germany

**Keywords:** Patient decision aids, Patient information, Shared-decision-making, Decision-making process, Therapy decision, Medical screening, Cancer, Oncological patients, Internet research

## Abstract

**Background:**

Previous studies have already shown that decision aids are a suitable tool for patient decision-making. The aim of this work is to conduct an online search for freely available, German-language patient decision aids (PDAs) for cancer patients, followed by an assessment of their quality. For this purpose, a rating tool that is as manageable as possible was developed on the basis of already existing quality criteria.

**Methods:**

A simulated patient online search was conducted via the four most frequently used search engines in Germany. A quality assessment tool was created utilizing international and national guidelines, with a focus on practicality and manageability. Subsequently, the identified PDAs were rated by 4 raters based on the rating tool.

**Results:**

The number of German-language oncology PDAs is low (n = 22 of 200 URLs) with limited variability regarding rare cancers. Most originate from non-profit organizations. The overall quality is low, as indicated by an average of 57.52% of the maximum evaluation points of the developed quality assessment tool. Reference values used to assess quality were related to e.g. support/effectiveness, adaptation, layout, etc. No qualitative differences were found regarding different publishers. Quality differed between PDAs of different length, with longer PDAs achieving better results.

**Conclusion:**

Overall, the supply and quality of German-language PDAs is not satisfactory. The assessment tool created in this study provides a solid, but more manageable basis, for developing and identifying high-quality PDAs.

**Practice implications:**

PDAs should be increasingly used by physicians in practice. For this, a quick qualitative assessment of PDAs in everyday life must be possible. Future research has to investigate especially the aspect of the length of a PDA in more detail.

**Supplementary Information:**

The online version contains supplementary material available at 10.1186/s12911-023-02259-4.

## Background

Cancer patients have a high need for information that provides insight into the progression and treatment options for their type of cancer [1–3]. Due to the increasing rates of new cases and the growing variety of complex therapy and detection methods, the “National Cancer Plan” was developed in Germany in 2008 by the Federal Ministry of Health in collaboration with other organizations. Two of the objectives set out in the project relate to improving the provision of information with the aim of making informed decisions and promoting participatory decision-making in line with the principle of “shared decision making” [4]. Patient decision aids (PDAs) are one way to implement these areas. PDAs are tools designed to support the weighing of personal decision options to facilitate the discussion of different treatment strategies and screening options between healthcare professionals and patients [5–7]. They provide evidence-based information on the various options with their associated advantages and disadvantages, as well as the probabilities of achieving a cure or detecting the disease. In this regard, PDAs should serve to prepare the patient to make an informed, value-based decision with his or her physician, thereby improving the match between the patient’s personal values and the options selected [5, 7–9]. In a Cochrane systematic review of decision aids related to health care treatments and screening procedures, it was found that patients who used these PDAs increased their participation in decision-making and improved their knowledge of the different options and realistic perception of outcomes. Furthermore, the authors concluded that the use of PDAs has had a positive impact on communication between physicians and patients and has not had a negative impact on health outcomes or patient satisfaction [3, 5, 6, 8, 9]. Considering the use of PDAs specifically in oncology for cancer patients, it can be stated from the survey in the 2021 paper by Josfeld, L. et al that patients who received a PDA were significantly more satisfied with the information provided. However, it was also determined that few patients received a PDA to assist in shared decision making. It was particularly clear in the survey conducted that the majority of these patients preferred shared decision making and that in doing so, the amount of information provided was overwhelming. According to the report by Josfeld, L. et al, PDAs have the potential to increase patient satisfaction and that their poor use requires easier access and better education about PDAs for clinicians [3].

PDAs are offered in the form of brochures, videos, computer programs, or on websites. In recent years, it has been observed that, in addition to the doctor’s consultation, the internet is being used as an increasing source of information [7, 10–12]. One problem is the large amount of information available, which is difficult for patients to assess in terms of quality and relevance [2, 3, 12]. Especially the discrepancy between visibility and quality can lead to misinformation and wrong decisions [13]. Especially internationally, the provision of PDAs on the internet is becoming increasingly popular due to its easy accessibility as well as low costs, so that more than 500 different PDAs could be registered [7, 9]. The Informed Medical Decisions Foundation in the USA and Great Britain is a pioneer in this field [14]. In addition, the International Patient Decision Aid Standards (IPDAS) Collaboration has existed since 2003 and in 2006 produced standards for quality criteria that can be used to improve the quality and effectiveness and evidence review of decision aids [7, 14].

With regard to the implementation of participatory decision-making, PDAs presented on the internet, and thus inexpensive, are of great importance. In light of this, Loh et al. compared eight European countries in their systematic review and found a greater disparity in Germany in terms of patient desire and actual participation. It was made clear that information on a wide range of treatment options with advantages and disadvantages was only communicated to a limited extent and that medical decisions were only made together with the patients in a few cases [9]. While these findings are older, improvement in patient participation still appears to be slow [15–18].

The aim of the present work is to investigate the quality, supply and availability of PDAs with regard to cancer in German-speaking countries by means of a simulated patient online search on the internet, and subsequently to develop an evaluation tool using the already existing international framework of quality criteria for PDAs to evaluate the PDAs with regard to their quality and effectiveness. This evaluation tool is to be based on the international standards. In order to create this tool, the various international guidelines are to be compared and weighed up, in particular which criteria could be especially relevant for German-language PDAs. In addition, the tool is to be supplemented by new criteria, if necessary. Furthermore, the tool is to be made compact and simple in terms of content, in order to optimize and facilitate handling in practice.

## Methods

### Procurement of the PDAs to be examined

**Fig. 1:**
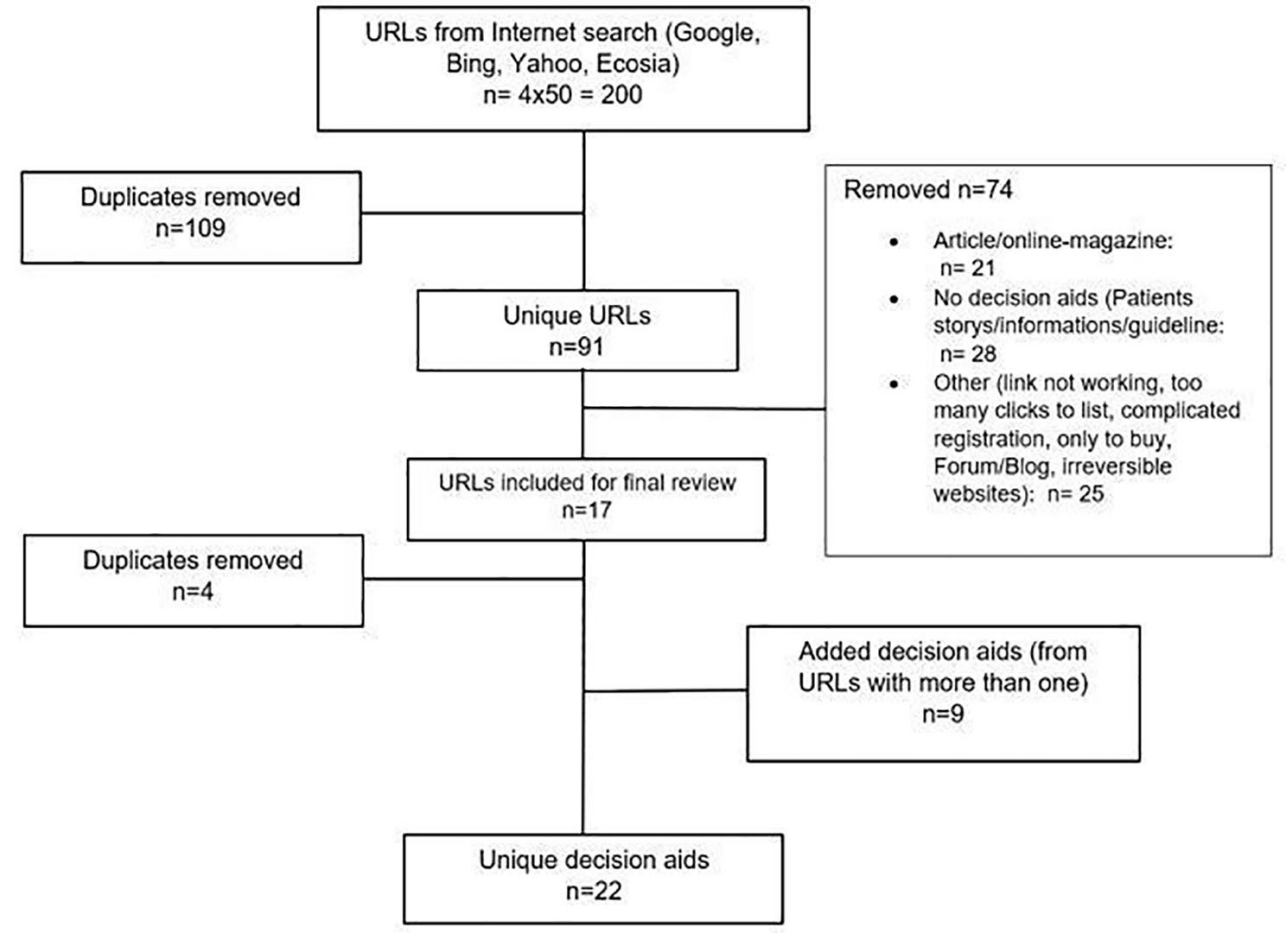
Process workflow of the website analysis

For this study, we simulated a patient’s online search for patient decision aids and then evaluated them using various content and formal criteria. The search was completed in October 2020 and followed the workflow as pictured in Fig. 1. We used the four most popular internet search engines within Germany in 2020 (Google, Bing, Yahoo and Ecosia), as classified by the “SEO-Summary” [19]. In each case, the first 50 results listed were searched for patient decision aids, or links pointing to another website with a PDA. As our research should mainly focus on PDAs connected to cancer, we used the phrase “decision aid cancer” (German: Patientenentscheidungshilfe Krebs) in the search bar. We excluded all patient decision aids unrelated to cancer. We tested this general procedure using the more specific search terms prostate cancer, breast cancer, colorectal cancer and lung cancer which are the four most important types of cancer and found no additional decision aids.

The selection of PDAs from all hits in the simulated search was done using defined inclusion and exclusion criteria (see Table 1) based on the IPDAS Collaboration definition (see Table 1) [7, 14]. Further exclusion criteria were aids for which the user would have to pay as well as PDAs hidden on websites that required more than five consecutive links to be followed to access them. The search and selection was done by JM and LJ.

**Table 1:** Inclusion and exclusion criteria for PDAs

Inclusion criteria	Exclusion criteria
Definition criteria must all be present	PDAs only against payment
Websites that link directly to decision aids	PDAs with complicated registration/club membership/subscription
**Definition criteria:**	Foreign-language (other than German) PDAs
- Describe the decision to be made and encourage active engagement with the decision-making process	More than 3 links to get to the PDAs (non-low-threshold)
- Provide evidence-based information about available medical options (including benefits, harms, and risks)	Complex guideline programs that would exceed the time frame of a consultation (not primarily declared as PDAs and not published as such)
- They make it clear that the patient’s goals, values and preferences play a role in the decision - according to the principle of personal decision-making	

### Development of our evaluation tool

To assess the PDAs for cancer patients we used the internationally listed IPDAS criteria developed by the International Patient Decision Aid Standards (IPDAS) Collaboration and the Quality Criteria Framework published by the International Online Delphi Consensus Process as a revision of the IPDAS criteria in 2006 [7, 20]. This two-stage web-based Delphi process, involving individuals from four stakeholder groups (researchers, practitioners, patients, policy makers) from 14 countries, involved an assessment of the importance of 80 criteria in 12 quality domains on a scale of 1 to 9 [7, 20].

To transform these criteria, which are meant for the development of PDAs, into a rating tool, we adapted a procedure we formerly have used to develop rating tools for other web-based patient information [21]. In these tools, criteria for patient information of the German Network for Evidence-Based Medicine, the Agency for Quality in Medicine, as well as HONcode, DISCERN, and afgis [22–26] have been merged to a basic set of criteria. This basic set may be specified for different types of patient information as websites [27], booklets [28], cancer apps [29]. To adopt the basic tool for German-language PDAs, we performed a defined process in three steps:

First, we added the 64 IPDAS criteria. Second, the individual criteria were compared and doublets eliminated. In the next step, we discussed overlapping criteria (JM; LJ) with the aim to formulate well-defined criteria. In case of dissent a third expert (JH) was included. The full development of our assessment tool can be seen in the E-Supplement as table S1.

**Fig. 2:**
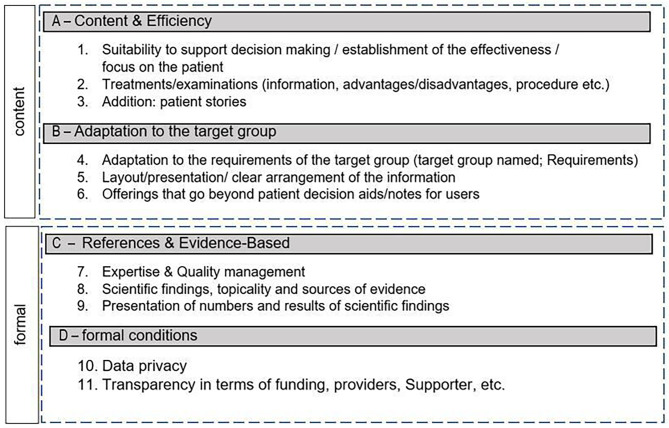
Categories for the evaluation of PDAs

This process resulted in an instrument which consists of 11 main categories with corresponding items (n = 42), which refer into two domains: content and formal (see Fig. 2). The final result of our evaluation tool can be viewed as document E2 in the E-Supplement. During the entire development process, the instrument was tested twice by three raters with a medical background on individual PDAs.

### Application of the evaluation tool

Consistent with the international online Delphi consensus process in revising the IPDAS criteria in 2006 and the 2015 Liebl et al. assessment tool, we also decided to use a rating scale to assess PDAs. For each item, 0 to 2 points could be assigned, with 2 points given for completely, 1 point for partially, and 0 points insufficiently fulfilling the respective criterion. Categories that were not applicable to a special PDA were marked as “not applicable” (n.a.).

For calculating the final score of each PDA, the scores of the various items within each category were summed and this sum divided by the maximum score, of all items in the category, thus eliminating disbalance by missing scorings for items not applicable to a single PDA. Each category could thus have a total score between 0 and a maximum of 2 points. In a final step, a total score was calculated for all categories in relation to the maximum achievable score, with scores ranging from 0 to 1. Then, this total score was expressed as a percentage.

The rating of decision aids was conducted by four independent evaluators (two physicians (CK, JH) and two medical students (JM, NZ)), using an Excel sheet.

### Statistics

For assessment and analysis, we used Microsoft Excel version of 2019. The Inter-rater concordance was calculated with IBM SPSS Statistics Version 27.0, using Kendall’s Coefficient of Concordance (Kendall’s W).

## Results

### Online findings

Following the online search, after removing duplications (n = 109) and applying the inclusion and exclusion criteria (n = 79 identified as no decision aids), a total number of 17 URL´s remained out of 200 web links that were utilized for a final review. Of these 17 URLs, another 4 web links had to be removed after the more detailed review, which contained or referred to the same PDA. Furthermore, nine URL’s were detected that contained more than one PDA or linked to multiple PDAs. Consequently, as described in Fig. 1, from the 13 URLs screened − 22 PDAs could be obtained for the subsequent evaluation. Of these, the majority emerged as products of non-profit organizations (n = 16, 72.73%), while PDAs from medical facilities/organizations (n = 5, 22.73%) and for-profit organizations (n = 1, 4.55%) comprised a smaller proportion. Most were listed as pdf files and were thus available as downloads (n = 17); the other part resulted from PDAs in the form of a website (n = 4) and as an interactive PDA (designed as a computer program, n = 1). The number of pages varied from 2 to 72 pages. In general, our selection of PDAs focused on screenings/medical examinations as part of a cancer screening (n = 12), medical treatments as part of cancer treatment (n = 6), or both (n = 4). Focusing on the different cancer types, the distribution was as follows: breast cancer (n = 8), colorectal cancer (n = 4), cervical cancer/HPV - human papillomavirus (n = 6), prostate cancer (n = 2), skin cancer (n = 1), chemotherapy in general (n = 1).

### Quality assessment

In this study, Kendall’s coefficient of concordance for the four raters was 0.663, indicating a reasonable level of agreement. The concordance between the two physicians was 0.61, and between the two medical students was 0.91. With respect to the criteria that related only to content, Kendall’s concordance coefficient for the four raters was 0.685 (between the two physicians = 0.707; between the two medical students = 0.852). For the Formal Criteria, a Kendall’s concordance coefficient of 0.521 was recorded for the four raters (between the two physicians = 0.447; between the two medical students = 0.874). In the overall ranking, the 22 PDAs achieved percentage scores ranging from 33.17% to 78.78%. On average, the PDAs reached 57.52% of the maximum rating score of the developed quality-assessment tool (SD = 13.25%). In the content/formal ranking, the PDAs were able to achieve a slightly better result for the content (M = 63.72%, SD = 14.38%) than for the formal criteria (M = 50.07%, SD = 14.66%).When looking at the evaluation of the individual main categories, clear differences could be noted. The highest average score was achieved by Part 2b: “screenings/medical examinations” (M = 83.00%, SD = 19.66%) - listed in category 2; whereas part 2a: “medical Treatments” (M = 56.95%, SD = 18.26%) was worse. The category “layout/presentation/ clear arrangement of the information” yielded the second highest value (M = 74.41%, SD = 25.81%) and could be evaluated for all 22 PDAs. The lowest values were achieved by “data protection” (M = 23.96%, SD = 30.03%), which was additionally applied to only 32.00% (n = 7) of the PDAs. “Adaptation to the needs of the target group” (22.73%, n = 5) was the least likely to be evaluated. Detailed results and the maximum and minimum ratings of each category are shown in Table 2.

**Table 2:** Rating results by category

Category	Mean	Median	Standard Deviation	Minimum	Maximum
*1.Suitability to support decision making / establishment of the effectiveness / focus on the patient (n = 22)*	49.73%	50.00%	20.72%	27.08%	85.42%
*2a) medical Treatments (n = 10)*	56.95%	64.59%	18.26%	29.17%	95.83%
*2b) Scrennings/medical examinations (n = 16)*	83.00%	93.00%	19.66%	39.00%	100.00%
*3.Addition: patient stories (n = 16)*	72.50%	71.88%	3.42%	68.75%	78.13%
*4.Adaptation to the requirements of the target group (n = 5)*	69.16%	71.88%	16.19%	0.00%	84.38%
*5.Layout/presentation/ clear arrangement of the information (n = 22)*	74.41%	87.50%	25.81%	0.00%	100.00%
*6.Offerings that go beyond patient decision aids/notes for users (n = 22)*	37.00%	34.00%	25.57%	0.00%	81.00%
*7.Expertise & Quality management (n = 22)*	47.41%	47.92%	22.57%	20.83%	79.17%
*8.Scientific findings, topicality and sources of evidence (n = 22)*	53.00%	58.00%	21.73%	0.00%	93.00%
*9.Presentation of numbers and results of scientific findings (n = 22)*	62.68%	75.00%	30.11%	8.33%	95.83%
*10.Data privacy (n = 7)*	23.96%	12.50%	30.03%	0.00%	87.50%
*11.Transparency in terms of funding, providers, Supporter, etc. (n = 22)*	35.24%	33.33%	14.65%	12.50%	62.50%
*Content rating (n = 22)*	63.72%	68.40%	14.38%	40.30%	80.80%
*Formal rating (n = 22)*	50.07%	52.73%	14.66%	22.38%	81.13%
*Overall rating (n = 22)*	57.52%	60.56%	13.25%	33.17%	78.78%

Placement in text: under paragraph: 3.2 Quality Assessment

### Qualitative differences between publishers

In Table 3, all the PDAs studied, as well as their origin and the percentages achieved for the overall ranking and the ranking in terms of content and formal aspects, are listed.

**Table 3:** Details and results of the individual PDAs.

	PDA´s	Type & length	Provider	Category of Provider	Total ranking	Content ranking	Formal ranking
1.	*Hormonrezeptiver-positiver Brustkrebs im Frühstadium. Eine Entscheidungshilfe für Frauen zur medikamentösen Behandlung.*	Pdf; 57p.	Gesundheitswissenschaften Universität Hamburg	Medical facilities/organisations	78.78%	70.33%	52.30%
2.	*Darmkrebs-Screening*	Pdf; 21 p.	Gesundheitswissenschaften Universität Hamburg	Medical facilities/organisations	76.44%	70.67%	53.30%
3.	*Früherkennung von Brustkrebs* *Eine Entscheidungshilfe für Frauen*	Pdf; 72p.	Arbeitsgemeinschaft der Wissenschaftlichen Medizinischen Fachgesellschaften e.V., Deutschen Krebsgesellschaft e.V. Deutschen Krebshilfe e.V.	Non-Profit-Organisation	76.10%	71.80%	42.40%
4.	*Früherkennung von Gebärmutterhalskrebs* *HPV-Impfung*	Pdf; 27 p.	BARMER	Non-Profit-Organisation	69.41%	78.33%	45.50%
5.	*Entscheidungshilfe Prostatakrebs*	interaktiv	DGU - Patientenakademie	Medical facilities/organisations	68.10%	80.80%	76.25%
6.	*Entscheidungshilfe Mammographie-Screening*	Pdf; 20 p.	Gemeinsamer Bundesausschuss	Non-Profit-Organisation	67.83%	67.50%	44.20%
7.	*Brusterhalt oder Brustentfernung? Eine Entscheidungshilfe für Frauen mit Brustkrebs, 7.Auflage 2018*	Pdf; 40 p.	AOK – Die Gesundheitskasse	Non-Profit-Organisation	63.41%	79.70%	56.50%
8.	*Gebärmutterhalskrebs Früherkennung -* *für Frauen zwischen* *20 und 34 Jahren*	Pdf; 20 p.	Gemeinsamer Bundesausschuss	Non-Profit-Organisation	62.78%	80.70%	70.38%
9.	*Brustkrebs Früherkennung* *Eine Entscheidungshilfe*	Pdf; 36 p.	Techniker KK	Non-Profit-Organisation	62.77%	59.00%	40.88%
10.	*Brustkrebs Früherkennung (Mammographie)*	Pdf; 19 p.	Barmer GEK	Non-Profit-Organisation	62.14%	49.20%	49.38%
11.	*Gebärmutterhalskrebs Früherkennung* *für Frauen* *ab 35 Jahren*	Pdf; 20 p.	Gemeinsamer Bundesauschuss	Non-Profit-Organisation	61.56%	40.30%	26.88%
12.	*Darmkrebs Früherkennung -* *Versicherteninformation* *für Frauen ab 50 Jahren*	Pdf; 24 p.	Gemeinsamer Bundesausschuss	Non-Profit-Organisation	59.56%	41.80%	22.38%
13.	*Entscheidungshilfe Mammographie-Screening*	Web	Barmer GEK	Non-Profit-Organisation	57.10%	75.40%	58.38%
14.	*Brustamputation –* *wie geht es weiter?* *Informationen und Entscheidungshilfen* *für Brustkrebspatientinnen*	Pdf; 48 p.	Frauenselbsthilfe nach Krebs Bundesverband e.V.	Non-Profit-Organisation	56.91%	63.60%	54.50%
15.	*Darmkrebs Früherkennung - Eine Entscheidungshilfe für* *Männer ab 50 Jahren*	Pdf; 11 p.	IQWiG	Non-Profit-Organisation	50.94%	69.00%	55.00%
16.	*Früherkennung Prostatakrebs* *PSA-Test: Sinnvoll oder nicht?* *Eine persönliche Entscheidungshilfe*	Pdf; 15 p.	AOK-Bundesverband, Universität Bremen, Krebsinformationsdienst des Deutschen Krebsforschungszentrums (DKFZ)	Medical facilities/organisations	49.28%	67.80%	53.80%
17.	*Früherkennung von Darmkrebs*	Pdf; 2 p.	KBV – Kassenärztliche Bundesvereinigung	Medical facilities/organisations	48.61%	45.00%	53.13%
18.	*HPV- Impfung*	Pdf; 2 p.	Barmer GEK	Non-Profit-Organisation	43.22%	47.80%	36.00%
19.	*Hautkrebsfrüherkennung*	web	TK – Die Techniker	Non-Profit-Organisation	42.56%	50.20%	28.25%
20.	*HPV-Impfung: Treffen Sie Ihre Entscheidung!*	web	TK – Die Techniker	Non-Profit-Organisation	40.44%	72.70%	81.13%
21.	*Früherkennung von Gebärmutterhalskrebs - eine Entscheidungshilfe*	web	TK – Die Techniker	Non-Profit-Organisation	34.33%	79.92%	56.80%
22.	*Chemotherapie – Eine Entscheidungshilfe*	Pdf; 4 p.	Biologische Krebsabwehr e.V.	For-profit organisations	33.17%	40.30%	44.13%
**Overall average**					**57.52%**	**63.72%**	**50.07%**
**Average non-profit-organisation (n = 16)**					**56.94%**	**64.18%**	**48.04%**
**Average medical facilities/organisations (n = 5)**					**64.24%**	**66.92%**	**57.76%**
**Average for- profit- organisation (n = 1)**					**33.17%**	**40.30%**	**44.13%**

Placement in text: under paragraph: 3.3 Qualitative differences between publishers

The three best rated PDAs ranked at overall values between 75 − 80% and were created by medical facilities/organizations (University of Hamburg; 78.78% & 76.44%) and one non-profit organization (AOK-Bundesverband, University of Bremen, Cancer Information Service of the German Cancer Research Center (DKFZ), 76.10%). Eight out of 22 PDAs were able to achieve a percentage value between 60 and 69.41%, the majority of which were published by non-profit organizations (n = 7) and one belonged to medical facilities/organizations. Values below 50.00% were given to a total of seven PDAs, which were also represented by non-profit organizations (n = 4) and medical facilities/organizations (n = 2). This includes the lowest rated PDA with 33.17% which was offered by a for-profit organization.

On average, non-profit organizations achieved a value of M = 56.94% (SD = 11.16%) and medical facilities/organization reached a value of M = 64.24% (SD = 14.25%). Compared to the for-profit organization (Only One: 33.17%), the ranking showed a higher difference in quality; however, all results should be interpreted with caution due to the small amount of data as well as the different distribution of PDAs based on publishers. The results in terms of content/formal ranking were similar to the overall ranking with only a slight difference (see Table 3).

### Qualitative differences in terms of the length

For the quality differences based on the size (number of pages) of the PDAs, the following classification was undertaken: short PDAs with 1–5 pages (n = 3), medium-length PDAs with 6–30 pages (n = 9), long PDAs over 30 pages (n = 5). Long PDAs achieved an average percentage of 67.59%, medium-length PDAs 62.22%, short PDAs 41.67%. This distribution can also be seen in Table 3, where PDAs with low page counts of 1 to 5 pages scored lower in the evaluation (ranks 22, 18, 17), while PDAs with the largest page counts (72 p. & 57 p.) scored in the top three. No major differences can be found in content/formal ranking based on the size, with content/formal: long PDAs M = 68.89%/49.32% (SD = 7.96%/7.18%), medium-length PDAs M = 62.81%/46.75% (SD = 15.17%/3.79%), short PDAs M = 44.37%/44.42% (SD = 14.67%/8.57%). The web-based PDAs (n = 4) were checked with regard to their length using the word count and are comparable to the medium-length PDAs. Due to the different format, these were not included in the calculation of the average values.

Based on the presentation/form of the PDAs, the web-based PDAs (n = 4), as opposed to the format: pdf file performed significantly worse, placing them primarily in the lowest ranks (ranks: 21,20,19,13). According to our evaluation, the interactive PDA was able to rank in 5th place. In this case, too, the small amount of data and the different distribution must be considered in the interpretation.

## Discussion

The aim of the present study was to investigate the quality and range of German-language PDAs for cancer patients and to develop a manageable evaluation tool for a quick assessment of quality in everyday practice. The first finding was obtained in the course of the online search simulation, where only 22 PDAs could be identified from 200 web links, indicating a relatively low supply of PDAs for cancer patients. With respect to the different cancer types on which PDAs focus, a limited variability is also evident. Past reviews have already noted that PDAs have a positive impact on decision-making, physician-patient communication, and knowledge of disease and related medical options [3, 5, 9, 14], underscoring the need to provide high-quality PDAs for cancer patients. Based on the rating, the average score for the PDAs was 57.52%, which corresponds to a generally low overall quality rating. Based on these results, we have to assume that high-quality PDAs for German-speaking cancer patients make up only a very small to nonexistent proportion or are significantly more difficult to find with regard to their accessibility and thus were not detected in the context of our online search.

Considering our evaluation tool, no established framework for quality standards directly related to German-language PDAs could be found in advance, so the international quality standards of the IPDAS Collaboration mainly served as a basis. While the IPDAS Collaboration focused on three main areas − 1. content; 2. development process; 3.effectiveness - our evaluation tool focuses on four main areas: A - Content & Efficiency; B - Adaptation to the target group; C - References & Evidence-Based; D - Formal conditions, which comprise a total of 11 categories in which the individual items were finally reworked and classified. International and German criteria catalogs were compared to develop a more compact instrument that can be more realistically used in practice by practitioners to assess the quality of PDAs [For a detailed list, please refer to Table S1]. Further, in the context of the development of future PDAs, the instrument should facilitate the overview of the various quality standards and thus help the authors to improve selected areas that have not yet been taken in to account. Due to the deterioration of health literacy in recent years, we considered the comprehensibility of a PDA to be particularly elementary [12]. Based on the IPDAS Collaboration, it was already established that a PDA should be understood by at least the majority of the target group. This point of view was intensified by our evaluation tool assuming that a PDA must be understood by all patients within the target group. Only thus can it act as a true support for physicians to clarify the most important information for a patient-oriented decision in an understandable way to patients in their daily practice during time-limited consultations.

In order for physicians to use or recommend PDAs in the future as information material or as a support during a patient consultation, a tool is needed that makes it possible to obtain an overview of the quality of the PDAs. One of several quality features of the IPDAS is the verification of the effectiveness during the development of the PDAs with corresponding field tests by their publisher/developer. This aspect was not included in our evaluation tool because we considered it difficult to verify without the corresponding studies and publications on hand, especially for people from a non-scientific background. Nevertheless, if this information were included in the PDA, it could provide the doctor with an indication of the efficacy and thus quality, so a respective item may be added to our evaluation tool at least as a supplement in the future.

PDAs specializing in cancer primarily include “medical examinations” in the form of screenings for the early detection of cancer, in addition to “medical treatments”. In the international quality standards, these are often summarized or listed as additional elements. Due to the sometimes lengthy and complex treatment procedures as well as the numerous screening examinations, we felt it was important to list these separately. In some cases, not all PDAs included both areas, but rather focused on one of the two topics, which made our separation appear advantageous.

The National Cancer Plan in Germany makes it clear that patient participation in decision-making processes and assumption of responsibility in therapy is nowadays already assumed (Goal 13 in the German National Cancer Plan) [4]. This expectation is supported by cancer patients’ ever-increasing need for information [4, 8]. Due to this, many PDAs are designed according to the principle of “shared decision-making”. In contrast, we did not want to focus exclusively on the “shared decision-making” approach, but added aspects that allow the patient to choose from all possible variants of decision making, including the preference for a decision made by the physician.

In our tool, eight out of eleven categories received at least once a mean rating of over 80%, the remaining three ranging between (31.82%) and (72.73%). Thus, high values can technically be achieved in every category. Also, the good agreement of the four raters speaks for our instrument, which leads us to the assumption that our established quality criteria are suitable for further use. They provide a framework for the preparation of future PDAs and for further quality and effectiveness studies. In contrast, our evaluation yields mediocre overall scores for content and form, so there is a need for optimization in these areas.

When looking at the individual categories, the category: “Layout/presentation/ clear arrangement of the information” received the best results. This could indicate that layout and presentation are of great importance in the creation of the PDAs, or that this category is much easier to fulfill than the content aspects. Additionally, it must be taken into consideration that the evaluation within this category might have been assessed differently if the group of evaluators had been extended to include the layperson’s perspective. Despite the fact that more and more emphasis has been placed on the protection of personal data in Europe in recent years, the category “data protection” is the lowest-rated category and was furthermore frequently classified as “not assessable”. One conclusion could be that it is not yet clear in the development of PDAs how this aspect is to be integrated or even designed. The same applies to the evaluation, which means that this category may often have been rated poorly, although it could have been classified as “not assessable” where no aspects of data protection were applicable. It is possible that this item was not sufficiently explained or defined in our tool as to what should be considered data protection. Thus, the different views of the evaluators and the level of information in this area may have contributed to this result. Other difficulties were encountered in evaluating the category “Adaption to the target group”. When creating our tool and in previous tests, we assumed that this aspect could in principle be used and evaluated for every PDA. However, this category was often associated with “not assessable” and we must therefore assume here that the associated content was not understood by the evaluators and would have to be explained in more detail.

Considering the origin of the PDAs, it can be seen that most of the PDAs were created by non-profit organizations - especially from statutory health insurance companies. However, based on our evaluation, there were hardly any quality differences between the PDAs from health insurance companies and those from medical institutions. Consequently, users cannot get an indication of the quality of the PDA based on the publisher alone, which reinforces the need for a tool to estimate the quality of a PDA.

Nevertheless, it is noticeable that all PDAs which were developed within the framework/participation of a university received the best quality ratings of the analyzed sample. It should be noted here that our evaluators all have a university background themselves, so these PDAs may have been rated better due to similarities between authors and evaluators regarding the important aspects of PDAs. Results might be different if based on a layman’s perspective.

Only one PDA could be assigned to the group of for-profit organizations, which also achieved the worst result. Since PDAs are mostly designed not to promote a specific product/medical treatment, but merely to support the patient by providing unbiased information, profit/income should be no focus when creating a PDA [6, 7, 14]. Therefore, it can be assumed that this area has not been of great importance for for-profit organizations so far. It should be mentioned here that PDAs, which are only available against payment or after subscription/registration to paid sites, were excluded from our study from the outset due to the difficulty of access.

Our study found considerable differences in length among the PDAs, with the shortest taking up two pages and the longest 72 pages. In this respect, no directives or recommendation as to the minimum or maximum length of a PDA could be found. Therefore, we must assume that this aspect has not yet been sufficiently considered or investigated in order to make an adequate statement and to evaluate PDAs in this respect. Nevertheless, shorter PDAs of lesser scope scored worse in terms of quality, while the most extensive PDAs had the best overall scores. In this context, the following aspects need to be further considered or clarified: The more extensive PDAs achieve qualitatively better ratings but could be more overwhelming for the reader. The longer versions, despite high ratings within the listed quality standards, may not meet the desired results in effectiveness and efficiency. On the other hand, the assumption that there are currently no high-quality PDAs of shorter length (1 to 5 pages) raises the question whether it is possible to create high-quality PDAs of shorter length with sufficient information. In the future, this aspect should be discussed or investigated with people from the relevant target group of PDAs.

### Limitations

First limitations arise from the self-imposed restrictions in the execution of the simulated patient online search (only German language, only 4 search engines, maximum of 50 transparent links per search, without specific search terms). In general, this study refers only to online searches (PDAs on the Internet), which according to the study by Hurrelmann et al. have become more relevant in recent years; especially among patients with long-standing chronic diseases [12]. Overall, a deterioration in health literacy was found in all age groups over the past six years, with older groups of people in particular showing higher deficits [12]. Based on this, it must be considered that offline material is still important for people with low digital health literacy, among others. This material was not included here, or only to a limited extent, where PDAs were available in both paper and digital versions.

Due to the focus on PDAs with cancer related- content, PDAs that specify other health-related topics were not included in our evaluation. Additionally, in order to achieve the largest possible selection, the applied search terms were chosen to be as non-specific as possible (“patient decision aid cancer”), which meant that any PDAs that would have resulted from a more specific search were not detected. Accordingly, these search terms, especially with a focus on specific cancer types, should be adjusted in a new study, to obtain more accurate results about what cancer patients would find online based on their specific cancer type. In our simulated online search, we looked for PDAs that focused on cancer, regardless of a specific stage within the disease. However, we found that the PDAs we detected and evaluated within our study focused almost exclusively on screening and prevention procedures related to cancer. A few referred to therapeutic procedures, but even these did not explicitly address advanced tumor stage. This should be taken in to account when interpreting our results.

Also excluded from our evaluation or not registered were PDAs which (1) were only available against payment or after login/registration on paid sites; (2) required more than three links to find (non-low-threshold); (3) were part of complex guideline programs that would exceed the time frame of a consultation (not primarily declared as PDAs and not published as such).In total, only a small amount of data was available with 22 PDAs, which must be taken into account when interpreting the results.

To evaluate the PDAs, an assessment tool was newly developed for this study, which was applied without prior implementation by a larger group of evaluators. Although the inter-rater concordance within the study seems appropriate and the tool was developed using internationally recognized quality standards, an evaluation still needs to be conducted especially given the variation between raters of different expertise. Also, certain items, in particular the item “data protection”, need to be specified in more detail within our evaluation tool in order to reduce discrepancies between the evaluators with regard to “assessable” or “not assessable” in the future. Furthermore, different categories were applied to a (sometimes vastly) differing number of PDAs, which may limit the comparability of the resulting values.

Since our evaluation instrument is primarily related to the handling in everyday practice by medical professionals, the evaluation was carried out by evaluators who all have a medical-academic background. An evaluation from the patient’s not carried out and should be included in future, point of view was therefore particularly with regard to the aspect of the comprehensibility of a PDA. This fact should also be taken into account in the interpretation.

## Conclusion

The clear majority of PDAs currently available for cancer patients do not represent a satisfactory result with respect to our quality criteria. To support the development as well as the evaluation of high-quality PDAs, a manageable tool is needed. From various international sources, there are already a large number of criteria that can be used as a basis in this regard. Since, despite the increasing presence of media such as the Internet, online portals, etc., the general practitioner still has the highest priority in health matters [12], it must be questioned whether some of these international quality standards are in part too complex and extensive and could therefore represent an obstacle in terms of time and content in everyday practice. A manageable tool not only serves to develop future PDAs, but can help physicians to provide optimal advice to patients in potentially time-limited situations and support them in their decision-making.

### Practice implications

The results of this study show that the supply and development of high-quality German language PDAs for cancer patients needs to be significantly improved, especially with regard to rare cancers. Since PDAs have a positive effect on the decision-making process and doctor-patient communication, they should be increasingly used by doctors in practice [3, 5, 6, 8, 9]. For this purpose, it is necessary that physicians can obtain a suitable overview of the quality of the PDAs available to them by means of a manageable instrument. The assessment tool developed in this study can be used as a basis for further research and elaboration of a suitable framework for quality standards regarding German-language PDAs in the future. Above all, the adaptation to the language level of the respective target group and to the progressive deterioration of health literacy [12] as well as educational level and social status should be taken in to account in the development of future PDAs.

An additionally important aspect is the length of a PDA, for which no recommendations could be found at present. Future studies should examine the scope of PDAs and provide guidance on the ideal length to achieve optimal results for patient-centered communication in everyday practice.

With regard to the development of future PDAs, patient-centeredness centeredness as well as the equal involvement of patients in the development process, should be given a high priority. In particular, this includes making the different types of decision-making clear to each patient in case the patient does not feel able or willing to act according to the principle of “shared decision-making”. Patient perspectives regarding PDAs in terms of efficacy and the various quality attributes should also be included in future research.

As long as it can be confirmed that PDAs contribute significantly to improving the decision-making process as well as to promoting the individual needs of patients, more should be done in these areas in the long term and, above all, the offer should be significantly expanded. This can be done, for example, by establishing an independent and publicly accessible registry that also contains quality information and guidelines for evaluating the PDAs listed there.

### Electronic supplementary material

Below is the link to the electronic supplementary material.

Supplementary Material 1

Supplementary Material 2

## Data Availability

The datasets used and/or analyzed during the current study are available from the corresponding author on reasonable request.

## Abbreviations

PDAs: Patient decision aids
URL: Uniform Resource Locator
IPDAS: International Patient Decision Aid Standards
HONcode (HON): Health on the Net
DISCERN: Qualitätskriterien für Patienteninformationen
afgis: Aktionsforum Gesundheitsinformationssystem
n.a.: not applicable
n: Number (Quantity)
M: Mean value
SD: Standard deviation
