# Supplementary material for: The quality of German - language patient decision aids for oncological patients on the internet
Source: BMC Med Inform Decis Mak. 2023 Aug 18;23:161. doi: 10.1186/s12911-023-02259-4 (PMC10436558; doi:10.1186/s12911-023-02259-4)
Supplement: Supplementary file 1 — Supplementary Material 1 [file 12911_2023_2259_MOESM1_ESM.pdf]

Assessment tool patient decision aids (PDAs) for cancer patients.

Rating: 0 = not fulfilled; 1= partly fulfilled; 2= fully fulfilled; n. a. = not assessable

A Content & Efficiency

1. Suitability to support decision making/establish efficacy with focus on the patient.

1.1 Helps to recognize that a personal decision needs to be made and guides one step by step through the decision making process, with the goal of getting closer to an individual decision.

1.2 Indicates that there are different options for the patient to arrive at a decision (Paternalistic - physician alone; Participative (PEF) - physician and patient; Informative - patient alone).

1.3 Helps to know about the available options or different features of the options and asks patients to think about which positive and negative features of the options are most important to them and to be clear about this at the end of the PDA.

1.4 Explains that personal values can influence decisions.

1.5 Provides an overall improvement in the match, between the options finally chosen and the values and characteristics that are most important to the patient.

1.5.1 Gives guidance to formulate the values with relevance to the EH
1.5.2 Refers to the decision whether the values should be included in the decision
1.5.3 Helps to implement the individual steps, in relation to the chosen decision form

1.6 The PDA describes various aspects of options to help patients imagine what it is like to experience the physical, emotional, and social effects of doing so.

2. a: For PDA to <u>treatments:</u>

2.1 In the PDA, the various treatment options are explained/shown to patients in a neutral and balanced manner.

2.2 The PDA provides information about the positive and negative features of each treatment (e.g., benefits or harms, side effects, disadvantages).

2.3 Includes the option to choose none of the treatment options [e.g., do nothing] and describes what happens in the natural course of a health condition if none of the listed treatments are chosen.

b: For PDA to <u>examinations</u>:

2.4 In the PDA, the various examinations are explained/shown to the patients in a neutral and balanced way. (e.g. what is done before, during and after the examination, as well as next steps based on the test results).

2.5 The PDA provides information about the positive and negative features of each test (e.g., benefits or harms, side effects, disadvantages), including obtaining a true/false positive and true/false negative test result.

2.6 Includes the option to choose none of the listed screenings [e.g., Do Nothing] and describes the odds of finding the disease with/without screening (e.g., diseases that would never have caused problems and thus would never be found without screening).

3. Addition (If patient stories are included in PDA).

3.1 Stories about other patients' experiences represent a range of both **positive and negative experiences,** illustrating how evaluations and decisions, for or against a potential option, vary.

3.2 The stories allow the patient to be able to relate to the **steps** others have taken **for their personal decision making** and use it for personal decision making.

3.3 Stories with other patients' experiences will be used to **introduce** the listed **options/measures** in the implementation/procedure as well as the possible outcomes to the target group.

3.4 **The steps used to select these stories** and the **steps experts** used to **verify** the information contained in these reports are identified. [in a reference section or accessible technical document].

B Adaptation for specific target groups

4. Adaptation to the needs of the target group

4.1 The PDA names/explains the target group for which it is intended
.

4.2 The PDA can be understood by all patients within this target group.

4.3 The PDA has low-threshold access for all patients within the target group at no additional cost

4.4 PDA offers options other than reading (e.g., audio, video or in -person discussion) to help patients

5. Layout aspects, presentation/clear arrangement of information

5.1 The PDA is clearly structured.

5.2 By using different design features (e.g. font, font color, font size), the PDA supports the content as well as the key messages and is thus able to neutrally compare/clarify positive and negative features of options. (e.g. same details with similar fonts etc.).

5.3 The PDA contains graphics/schematics/visual diagrams that reflect/transport the content of the decision aid and do not distract from the core topic.

5.4 For PDAs on the Internet:

- **5.4.1** Provides a step-by-step way to move through web pages (screens) on the Internet.
- **5.4.2** Patients can search for keywords in the decision aid.
- **5.4.3** Make it easy for patients to return to decision support after linking to other websites.
- **5.4.4** Can also be printed as a single document (e.g. pdf document)

6. Offerings/instructions for users that go beyond the patient decision aid

6.1 The PDA includes tools such as worksheets or checklists that can be used when discussing options with a practitioner.

6.2 Web-based PDAs include the ability to provide feedback on personal health information that can be included in patient decision support. (e.g., web pages with questionnaires or similar).

C References & Evidence-Based

7. Expertise & Quality Management

7.1 The development of the PDA included the review of external experts [health professionals] who were not involved in the development or field trials, or a test, with on-site users adapted to the target group.

7.2 Contains the developer data and credentials, as well as the qualifications and references about the people who developed them.

7.3 Patients/practitioners were asked in advance what they needed to prepare them for a specific decision/what they needed to discuss a specific decision with patients.

8. Scientific evidence, timeliness, and sources of evidence

8.1 Contains references to scientific findings

8.2 Contains the steps for selecting the scientific evidence (e.g., find, claim, summarize) are included [in a reference section or accessible technical document].

8.3 Indicates the date it was last updated and how often the information in the decision aid is updated.

8.4 Describes the quality of scientific knowledge/evidence (e.g., quality of research studies) [including lack of evidence].

8.5 Includes references (e.g., citations) for the evidence listed and uses data from studies of patients similar to those in the target population (e.g., age, sex, risk group, disease).

9. Observance of scientific knowledge about the presentation of figures and results

9.1 Presents probabilities/event rates related to patients within the appropriate target group (e.g., age, risk group, same disease) and compares outcome probabilities using the same denominator, time period, and scale.

9.2 Describes the uncertainty around probabilities (e.g., by giving a range or using phrases such as "our best guess is") and puts probabilities in context with other events (e.g., chances of developing other diseases, dying from other diseases, or dying from any cause; with survival and mortality rates).

9.3 Describes how probabilities were calculated (in a reference section or accessible technical document), and if disease probabilities of subgroups [e.g., younger, middle-aged, or elderly] are provided, describes the tool used to estimate those risks.

D Formal framework

10. Privacy

10.1 Provides security for own personal health information included in PDA (e.g., on websites).

10.2 When stories are used in a PDA, a statement is included that patients have given informed consent to have their stories included. [in a reference section or accessible technical document].

11. Transparency in terms of providers, supporters, funding, etc.

11.1 Indicates the funding source for the development and distribution of the PDA.

11.2 The PDA indicates whether the originators, or author of the decision aid, stand to gain or lose by the decisions patients make after applying a decision aid.

11.3 If the PDA includes stories about other patients' experiences, it reports whether there was a financial or other reason why patients decided to share them.
