# Supplementary material for: The quality of German - language patient decision aids for oncological patients on the internet
Source: BMC Med Inform Decis Mak. 2023 Aug 18;23:161. doi: 10.1186/s12911-023-02259-4 (PMC10436558; doi:10.1186/s12911-023-02259-4)
Supplement: Supplementary file 2 — Supplementary Material 2 [file 12911_2023_2259_MOESM2_ESM.pdf]

Table S1: Comparison of evaluation criteria of patient decision aids and the criteria for evaluating websites, in the analysis of evidence-based patient information of cancer patients.

**IPDAS 2005** (criteria for assessing quality for patient decision aids).	Similarities between the evaluation criteria	**Paper: Developing a quality criteria framework for patient decision aids: online international Delphi consensus process;** 26. August 2006<br>https://www.ncbi.nlm.nih.gov/pmc/articles/PMC1553508/	Similarities between the evaluation criteria	**What cancer patients find on the internet - the visibility of evidence-based patient information Analysis of information on German websites** (criteria for the evaluation of websites)
				**1.** criteria for the evaluation of the Information quality
**1.1** Includes peer review with patients/subject matter experts who were not involved in developing and are involved in the field tests as independent experts	←	**1.1** The decision aid was reviewed by external experts [health professionals] who were not involved in the development or field trials	←	**1.1**<br>•Expertise - a report written by experts on a specific issue in a field of expertise.
**1.1.1**		**1.1.1**		**1.1.1**
• Contains procedures for quality assurance of a scientific paper or project by independent reviewers from the same discipline<br><br>• Will be field tested with users (patients facing decision; practitioners presenting options)	←<br><br>←	• The decision aid was reviewed by external experts [patients who had previously faced the decision] or health professionals who were not involved in the development or field trials<br><br>• Practitioners counseling patients on the option field tested the decision aid.<br>• Patients who faced the decision field tested the decision aid	←	• Quality management

• Contains the developer credentials/qualification<br>s	←	• The patient decision aid contains information about the credentials of the people who developed it<br>• Patients were asked what they needed to prepare them to make a particular decision<br>• Practitioners were asked what they need in order to discuss a specific decision with patients		
				**1.2** Explanation of the objectives and Target groups<br>**1.2.1** Achieving the goals<br>**1.2.2** Relevance
**1.3**		**1.3**		**1.3**
Field testing with users [patients, practitioners] indicates that the Patient Decision Aid:<br>•Reasonable/justifiable is<br>•Balanced/neutral for undecided patients is	←	• Field testing showed that decision support was acceptable/appropriate for patients<br>• Field testing showed that decision support was acceptable/appropriate for practitioners<br>• Field tests showed that undecided patients felt that the information was presented in a balanced manner	←	•Fair balance/ neutrality

**1.4 Use simple language?**		**1.4 Using simple language**		**1.4**
		• The patient decision aid identifies the reading level at which it is written and the formula [method] used to determine the level	←	• Comprehensibility for layman (e.g. suitable graphics)<br>• Language adapted to the needs of the target group
• Is written at a level that can be understood by the majority of patients in the target group	←	• The patient decision aid is written at a level that can be understood by at least half of the patients for whom it is intended		
• After the readability assessment, is written at a level equal to or below grade 8 (SMOG or BRJ).	←	• The patient decision aid is written at a level no higher than grade 8 [or equivalent] according to a readability formula (e.g., SMOG or BRJ)		
• Provides ways to help patients understand information other than reading [audio, video, face-to-face discussion].	←	• Patient decision support provides ways to help patients understand information other than reading (e.g., audio, video, or in-person discussion)		
• Can be understood by people with limited reading skills	←	• Field tests showed that the patient decision aid was understood by patients with limited reading skills		
		• The patient decision aid describes the "professional standards for plain language materials" that guided its development (e.g., Plain Language Association International)		

1.5 Decision-making processes leading to Lead decision quality		1.5 Establishing effectiveness		1.5
• Recognize/show that a decision needs to be made.<br>• Be clear about the option features that are most important at the end of the patient decision aid<br>• Request to discuss options with a physician<br>• Mention that personal values influence decisions<br>• Involve patients in the decision<br>• Improving the match, between the existing and finally chosen options with the values and characteristics that are most important to the patient.	←<br>←<br>←<br>←<br>←<br>←	• Helps to recognize that a decision must be made<br>• Helps to know about the available options<br>• Helps to know about different features of the options<br>• Helps to be clear about which features of options are most important to them<br>• Helps to discuss values with their doctors<br>• The patient decision aid suggests ways to talk to a doctor about the decision<br>• Helps to understand that values influence decision making<br>• Helps to participate in decision making in their preferred way<br>• The match between the functions that are most important to the informed patient and the option chosen improves	←	• Suitability to support joint decision making

**1.6 Use current scientific evidence -** cited in a reference section or technical document.<br>become?		**1.6 Based on current scientific findings**		**1.6**
• Includes references to the evidence used	←	• Contains references to scientific findings	←	• Scientific findings and topicality<br>• No statements on topics without evidence<br>• Marking missing evidence<br>• Completeness of information on sources of evidence<br>• Information on additional references and resources<br>• Accuracy/thoroughness
• Report steps to search, evaluate, summarize evidence.	←	• The steps for selecting scientific evidence (e.g., find, claim, summarize) are included [in a reference section or accessible technical document]		
• Report date of the last update<br>• Report how often the patient decision aid is updated	←	• Indicates the date when it was last updated<br>• Specifies how often the information in the decision aid is updated		
• Describe the quality of scientific evidence (including missing evidence).	←	• Describes the quality of scientific knowledge (e.g. quality of research studies)<br>• Describes the quality of scientific evidence (e.g., quality of research studies) [including lack of evidence].		
• Uses evidence from studies with patients similar to those in the target population	←	• Uses evidence from studies of patients similar to those who would use decision support (e.g., age, sex)		

1.7 Provide information about options in sufficient detail for the decision-making process?		1.7 Providing information about the possibilities/options		1.7
• Describe the state of health<br>• Options list<br>• List the possibility of doing nothing<br>• Describe the natural course without options<br>• Describe procedures<br>• Describe positive features (advantages)<br>• Describe negative features of options [damage / side effects / Disadvantages]<br>• Describe which test is designed for the measurement<br>• Include opportunities for positive / negative outcomes<br>• Chances of real positive, real	←<br>←<br>←<br>←<br>←<br>←<br>←	• The patient decision aid describes the health condition in the context of the decision to<br>• Patient decision aid lists health care options<br>• The option not to select any of the health options [e.g. Do nothing] is included<br>• The patient decision aid describes what happens in the natural course of a health condition when none of the health care options is chosen<br>• The patient decision aid includes information about the procedures involved (e.g., what is done before, during, and after the health option)<br>• The patient decision aid provides information about the positive features of the options (e.g., advantages, benefits)<br>• The patient decision aid has information about the negative characteristics of the options (e.g., harms, side effects, disadvantages)<br>**For decision support that focuses on testing**<br>• Has information about what the test is supposed to measure<br>• Has information about the chances of getting a true positive, true positive, false positive, and false negative test result. to obtain	←	• Detailed information about the treatments,<br>• their benefits and risks,<br>• Impact on quality of life;<br>• Mode of action;<br>• Consequences of non-treatment

negative, false positive, false negative test results<br><br>• Describe possible next steps based on test result<br><br>• Include chances that the disease will be found with / without screening<br><br>• Detection description /treatment that would never have caused problems if you were not screened.	←<br><br>←<br><br>←	• Describes possible next steps based on the test results<br><br>• Has information on the chances of diseases being found with and without screening<br><br>• Provides information on the detection and treatment of diseases that would never have caused problems if screening had not been performed		
**1.8 Does it include methods for clarifying and expressing patient values?**		**1.8 Clarification and expression of values**		**1.8**
• Describe procedures and outcomes that help patients imagine what it is like to experience their physical, emotional, social impact<br><br>• Ask patients to consider which positive and negative features are most important<br><br>• Suggest ways patients can share what matters most with others<br><br>• Feedback on personal health information that can be included in patient decision aid	←<br><br>←<br><br>←	• The patient decision aid describes the characteristics of options that help patients imagine what it is like to experience their physical, emotional, and social impacts<br><br>• The patient decision aid asks patients to think about which positive and negative features of the options are most important to them<br><br>• Patient decision support suggests ways patients can share what matters most to them when others are involved in decision making	←	• Focus on the patient

**1.9 Information in balanced way presented?**		**1.9 Weighing the presentation of options** Patient Decision Support...		**1.9**
• Able to compare positive and negative characteristics of options<br>• Shows negative / positive features with the same detail (fonts, sequence, display of statistics)	←<br>←	• Allows to compare the positive and negative features of the available options<br>• Shows the negative and positive features of options with the same details (e.g. with similar fonts, order, display of statistical information)	←	• Layout aspects<br>• Clear arrangement of the information<br>• Completeness
				**2. formal criteria**
**2.1 Disclose conflicts of interest?**		**2.1 Disclosure of conflicts of interest** Patient Decision Support...		**2.1**
• Funding source for development and distribution of patient decision aids<br>• Report whether authors or their affiliations stand to gain or lose from the decisions patients make after using patient decision aids<br>• Reports if there was a financial or other reason why patients decided to share their story	←<br>←<br>←	• Reported where the money came from to develop the decision support tool<br>• Reported where the money came from to copy and distribute the decision aid<br>• The patient decision aid reports whether the originator or author of the decision aid stands to gain or lose from the decisions that patients make after using a decision aid<br>• If the patient decision aid includes stories about other patients' experiences, it reports whether there was a financial or other reason why patients decided to share them	←	•Transparency in relation to Vendors, supporters, funding, advertising, etc.

**2.2 Data protection**		**2.2**		**2.2**
• Provides security for personal health information included in the decision support tool<br><br>**When stories are used in patient decision support**<br>• State in an accessible document that the patient has given informed permission for their stories to be used		• When stories are used in a patient decision aid, a statement that patients have given informed consent to have their stories included. [in a reference section or accessible technical document].		• Privacy
**2.3 Present the Probability of results in an unbiased and understandable way?**		**2.3 Presenting probabilities**		**2.3**
•Use event rates that indicate the population and time period<br><br>• Compare outcome probabilities using the same denominator, time period, and scale<br><br>• Using visual diagrams<br><br>• Use multiple methods to display probabilities [words, numbers, graphs].		Patient Decision Support...<br>• Represents probabilities using event rates in a defined patient group for a given time<br>• Compares the probabilities of options with the same denominator<br>• Compares the probabilities of options in the same time period<br>• Uses visual diagrams to display the probabilities (e.g., areas, stick figures, or bar graphs)<br>• Uses the same scales in the diagrams where options are compared<br>• Provides more than one way to explain probabilities (e.g., words, numbers, diagrams)		• Observation scientific knowledge about the presentation of figures and results

• Allow patients to consider probabilities based on their own situation (e.g., age, risk group, etc.)	←	• Enables patients to recognize the probabilities of what might happen based on their individual situation (e.g., specific to their age or severity of illness)<br>• Allows patients to select a way to display probabilities (e.g., words, numbers, graphs)		
• Description of the uncertainty around probabilities	←	• Describes the uncertainty around the probabilities (e.g., by giving a range or using phrases like "our best guess is")		
• Putting probabilities in context with other events	←	• Places emphasis on the odds of what might happen in other situations (e.g., odds of developing other diseases, dying from other diseases, or dying from any cause)		
• Both positive and negative frames are used [e.g., with survival and death rates].	←	• Represents probabilities that include both positive and negative frames (e.g., with survival and mortality rates)		
		• The way in which the probabilities were calculated is described (in a reference section or an accessible technical document)<br>• If the likelihood of disease of subgroups [e.g., younger, middle-aged, or elderly] is provided, the tool used to estimate these risks is described [in a reference section or accessible technical document]		

**2.4 Structured consulting and Include communication?**		**2.4 Leadership/Coaching in Consulting and Communication**		**2.4**
• Steps for decision making<br>• Suggestions on how to talk to a doctor about the decision<br>• Include tools [worksheet, question list] to discuss options with others	←<br>←<br>←	• The patient decision aid explains a step-by-step process to make a decision<br>• The patient decision aid suggests ways to talk to a doctor about the decision<br>• Patient decision support offers the opportunity to work with a trained "coach" to help patients consider their options<br>• Patient Decision Support offers the opportunity to work with a trained "coach" to help patients prepare to make decisions with a physician<br>• Patient decision support includes tools such as worksheets or lists of questions that can be used when discussing options with a practitioner	←	• Possibilities of the Feedback and participation for users
**2.5 Facilitation on websites**		**2.5 Provision of Patient decision aids on the Internet**		
• Providing a step-by-step way to go through the web pages<br>• Enable patients to search for keywords	←<br>←	• Provides a step-by-step way to move through the web pages (screens) on the Internet<br>• Patients can search for keywords in the decision aid		

• See point privacy<br>• Make it easier for patients to return to decision support after linking to other websites<br>• Allow printing as single document	←<br>←<br>←	• Provides feedback on personal health information entered into the decision aid. [e.g., the chances that you will have a complication].<br>• Does the website provide security for personal health information entered into the decision support tool<br>• Is it easy for patients to find their way back to where they were in the decision support when they clicked on links to other websites<br>• If the patient decision aid is on the internet, it can also be printed as a single document (e.g. pdf- document)		
**2.6 Meet additional criteria when stories are used in patient decision support become?**		**2.6 Decision support with patient stories**		
• Use stories that represent a range of positive and negative experiences	←	• Gives stories about the experiences of other patients<br>• Do the stories represent a range of experiences (positive and negative)<br>• If the steps used to select these stories are described [in a reference section or accessible technical document].<br>• If the steps experts used to verify the information contained in these reports are included [in a reference section or accessible technical document]		
